# MoS_2_‐Coupled Carbon Nanosheets Encapsulated on Sodium Titanate Nanowires as Super‐Durable Anode Material for Sodium‐Ion Batteries

**DOI:** 10.1002/advs.201900028

**Published:** 2019-03-22

**Authors:** Shitong Wang, Fangjun Cao, Yutong Li, Zhongtai Zhang, Daming Zhou, Yong Yang, Zilong Tang

**Affiliations:** ^1^ State Key Lab of New Ceramics and Fine Processing School of Materials Science and Engineering Tsinghua University Beijing 100084 China; ^2^ Department of Nuclear Science and Engineering Massachusetts Institute of Technology Cambridge MA 02139 USA; ^3^ College of science Northwest A&F University Yangling Shaanxi 712100 China; ^4^ State Key Lab of Solidification Processing Center of Advanced Lubrication and Seal Materials Northwestern Polytechnical University Xi'an Shaanxi 710072 China; ^5^ School of Astronautics Northwestern Polytechnical University Xi'an Shanxi 710072 China

**Keywords:** core–shell, MoS_2_ nanosheets, sodium titanate nanowires, sodium‐ion batteries, strong‐coupled carbon

## Abstract

There is an ever‐increasing demand for rechargeable batteries with fast charging, long cycling, high safety, and low cost in new energy storage systems. Herein, a heterogeneous architecture composed of MoS_2_‐coupled carbon nanosheets encapsulated on sodium titanate nanowires is developed and demonstrated as an advanced anode for sodium‐ion batteries (SIBs). Owing to the synergistic effects of ultrastable substrate of 1D sodium titanate (NTO) nanowires, high‐capacity promoter of 2D MoS_2_ nanosheets as well as the 2D conductive carbon matrix, the resulting 1D/2D–2D hybrid demonstrates excellent high‐rate capacity and super‐durable cyclability, delivering a stable capacity of up to 425.5 mAh g^−1^ at 200 mA g^−1^. Even at an ultrafast charging/discharging process within 80 s, the capacity can be maintained at 201 mAh g^−1^ after 16 000 cycles with only 0.0012% capacity loss per cycle, one of the best high‐rate capacities and cyclabilities for NTO‐based hybrid composites. The present work highlights the designing protocol of hierarchical nanoarchitectures with stable substrate and high‐capacity electrodes for next‐generation energy storage applications.

With the rapid development of electronic vehicles (EVs) all around the world, the limited lithium reserves could not support the huge revolution process from fuel vehicles to EVs.^1^ As an alternative to lithium‐ion batteries (LIBs), sodium‐ion batteries (SIBs) are receiving growing attention owing to the abundant resources of sodium element (≈2.64% of the earth's crust) and its similar physical and chemical properties to lithium.^2^ Among various electrodes of SIBs, layered sodium titanates (NTO) with a formula of Na_2_Ti*_n_*O_2_ *_n_*_+1_ (2 ≤ *n* ≤ 9), such as Na_2_Ti_3_O_7_,^3^ Na_2_Ti_6_O_13_,^4^ Na_2_Ti_9_O_19_,^5^ have been considered as ideal anodes because of their low potential (<1.0 V vs Na/Na^+^) and relatively large interlayer spacing (7–9 Å) for ionic transport. However, the insulating nature and relatively sluggish reaction kinetics limit the electrochemical performances of layered sodium titanates. Several strategies have been adopted to solve those shortcomings, including nanostructuring, hydrogenation, and compositing with carbon materials.^[3a,c,4b,6]^ Compared with bulk materials, nanosized materials, especially 1D nanostructure, can shorten ionic diffusion path, improve the contact area between electrode and electrolyte, as well as relieve the stress caused by structural volume expansion during electrochemical reactions, leading to high‐rate capacity and cyclability enhancement.^7^ Recently, 1D NTO materials have been demonstrated as a promising candidate for SIBs.[qv: 3c,4b,6b,8] Except for the electronic and ionic conductivity, relatively low gravimetric/volumetric energy density is another critical issue for layered sodium titanates. Therefore, combining with high‐capacity active materials is an efficient strategy to solve the Achilles' heel.^9^

Layered MoS_2_, as a typical conversion‐type anode, has been widely studied for high‐capacity Li^+^ and Na^+^ storage.^2^, ^10^ However, the huge volume variation during electrochemical process leads to serious pulverization and poor cyclability. Particularly, it has been demonstrated that decreasing the conversion‐type materials into the nanometer range is an efficient way to alleviate or reduce the stress caused by volume change, whereas the cyclability is still unsatisfactory due to aggregation and pulverization of nanostructure. Recent progress reveals that excellent electrochemical performances can be achieved by designing hierarchical structures.[qv: 10f,11] Just as mentioned above, layered sodium titanates exhibit tiny structural changes in the electrochemical sodiation/desodiation process, leading to excellent high‐rate and long‐cycling performances. Consequently, rational constructing the synergistic effects of highly stable substrate of sodium titanates, the high‐capacity feature of MoS_2_, as well as conductivity enhancement with some carbonaceous materials is advisable for ideal advanced anodes, but it is still a great challenge.

In this work, we rationally designed and synthesized a hierarchical core/shell nanoarchitecture composed of 1D NTO nanowires core and MoS_2_ nanosheets coupled with carbon nanosheets. The as‐resulting core/shell NTO/MoS_2_‐C composite possessed multiple structural features. For one thing, the 1D layered sodium titanates act as stable core substrates and the epitaxially aligned MoS_2_–carbon coupled interface nanosheets can effectively enlarge the capability of Na^+^ storage; for another, external 2D carbon heterogeneous structures not only can prevent the aggregation of 1D NTO substrates and high‐capacity promoter MoS_2_, but also improve the electronic conductivity of the whole nanoarchitectures. As a result, the nanocomposites exhibit significantly enhanced sodium ion storage compared to pure NTO nanowires. Qualitative leap in specific capacities shows about 10 times improvement at 200 mA g^−1^, and 30 times improvement at 8000 mA g^−1^. Even at ultrafast charging/discharging process within 80 s (8000 mA g^−1^), the novel architecture can sustain more than 16 000 cycles with merely 0.0012% capacity loss per cycle, demonstrating it could be an ideal high‐rate and long‐cycling host for SIBs. This work would give an enlightenment by designing hierarchical architectures with stable substrates and high‐capacity electrodes for next‐generation energy storage applications.

**Figure** **1** illustrates the synthetic procedure involving the following three steps. First, 1D hydrated sodium titanate nanowires precursor (denoted as Na_2_Ti_2_O_5_·H_2_O) substrate can be synthesized by a simple hydrothermal process.^5^, ^8^ As shown in Figure S1 (Supporting Information), the 1D Na_2_Ti_2_O_5_·H_2_O nanowires exhibit a large length‐diameter ratio. Then, heterogeneous architectures assembled by 1D Na_2_Ti_2_O_5_·H_2_O/2D‐Mo‐polydopamine (PDA) nanosheets (denoted as Na_2_Ti_2_O_5_·H_2_O/Mo‐PDA) can be fabricated by a polymerization process (Figure S2, Supporting Information), and the 2D–2D Mo‐PDA were densely packed on the surface of 1D Na_2_Ti_2_O_5_·H_2_O substrate. Finally, phase transformation and carbonization processes were performed via thermal treatment. During this process, the core Na_2_Ti_2_O_5_·H_2_O nanowires converted into sodium titanate, and the external Mo‐PDA nanosheets simultaneously transformed to MoS_2_ nanosheets and conductive‐carbon nanosheets, leading to the formation of core/shell hybrids (denoted as NTO/MoS_2_‐C).

**Figure 1:**
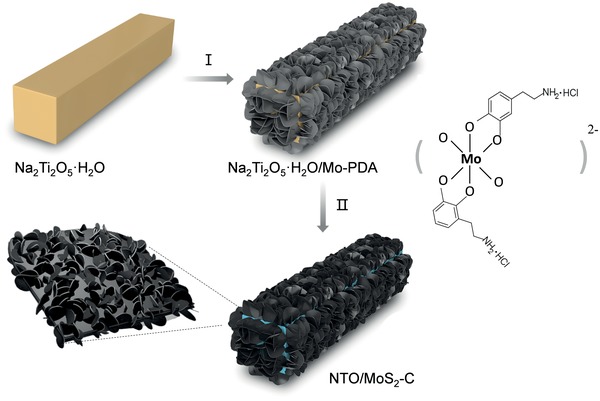
Schematic illustration of the fabrication procedure of NTO/MoS_2_‐C nanoarchitectures (I: epitaxially growth process, II: calcination conversion process).

The phase composition of NTO/MoS_2_‐C was initially analyzed by X‐ray diffraction (XRD). As shown in **Figure** **2**a, the diffraction peaks correspond well to the monoclinic Na_2_Ti_9_O_19_ phase (JCPDS No. 33‐1293) and MoS_2_ phase (JCPDS No. 75‐1539). There are no obvious signals about other species. It is proposed that the 2D carbon precursor is grown on the surface of NTO during the polymerization process, and then convert to conductive carbon nanosheets during calcination process. According to elemental analysis from inductively coupled plasma mass spectroscopy (ICP‐MS), the contents of MoS_2_ and NTO in NTO/MoS_2_‐C are 33.4 and 41.3 wt%, respectively. It is rational to believe that the content of the carbon species is about 25.3 wt%. In addition, typical D peak (1360 cm^−1^) and G (1560 cm^−1^) peaks in Raman spectroscopy analysis (Figure S3, Supporting Information) further indicate an amorphous feature of the carbon structure.

**Figure 2:**
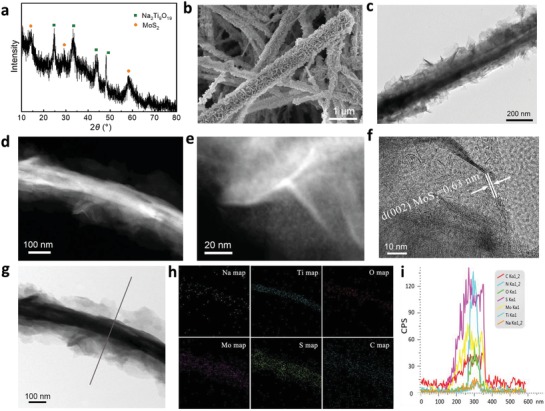
Characterization of NTO/MoS_2_‐C nanoarchitectures. a) XRD pattern, b) SEM image, c) TEM image, d,e) STEM images, f,g) HRTEM image as well as h) mapping and i) linear scanning analysis of NTO/MoS_2_‐C.

The heterogeneous morphology and structure of NTO/MoS_2_‐C were characterized by scanning electron microscopy (SEM) and transmission electron microscopy (TEM). It is clearly to see that rambling nanosheet‐like structures are uniformly grown around the NTO nanowires (Figure 2b,c). Scanning transmission electron microscopy (STEM) diagrams (Figure 2d,e) and high‐ resolution TEM (HRTEM) diagrams (Figure 2f,g and Figure S4, Supporting Information) clearly illustrate that numerous sheet‐like structure with a thickness of about 3–5 nm is well aligned and uniformly dispersed on the surface of 2D carbon conductive nanosheets, forming a special 2D–2D architecture. At higher resolution of HRTEM, the interplanar spacings of MoS_2_ nanosheets and NTO substrate can be detected clearly: 0.63 nm corresponds to the (002) crystal planes of MoS_2_ (Figure 2f); 0.36 and 0.50 nm associate with the (110) and (201) crystal planes of Na_2_Ti_9_O_19_, respectively (Figure S5, Supporting Information). Linear scanning analysis (Figure 2g) and the corresponding energy disperse spectroscopy (EDS) mapping analysis (Figure 2h) show that the MoS_2_ nanosheets are uniformly distributed in the carbon nanosheets layer and MoS_2_‐C layer is grown on the surface of NTO substrate. The corresponding EDS linear scanning analysis (Figure 2i) illustrates that the inner core substrate is mainly composed of Na, Ti, and O elements. It is worth mentioning that the external MoS_2_‐C nanosheets formed on the NTO nanowires are highly uniform and stable. Obviously, this core–shell structure can not only greatly improve the electronic conductivity of the MoS_2_ and the NTO substrates, but also can enhance the wettability of the whole nanoarchitectures in the electrolyte; more importantly, it also helps to alleviate the volume change and agglomeration of the MoS_2_‐C nanosheets during charge and discharge, which maintains the structural integrity.

The surface chemical state and composition of NTO/MoS_2_‐C materials can be revealed by X‐ray photoelectron spectroscopy (XPS). In XPS full spectrum (**Figure** **3**a), the peaks located at around 163 eV (S 1s), 230 eV (Mo 3d), 285 eV (C 1s), 400 eV (Mo 3p), 460 eV (Ti 2p), and 532 eV (O 1s) indicate that the NTO/MoS_2_‐C is composed of Na, Mo, S, Ti, C, and O elements. The Mo 3d_3/2_ (232.8 eV) and 3d_5/2_ peaks (229.6 eV) are both consistent with the Mo (IV) oxidation state of MoS_2_ 12 (Figure 3b). The small peak located at 226.2 eV is assigned to S 2s of Mo–S (Figure 3c). Besides, the binding energies of S 2p_3/2_ (161.8 eV) and S 2p_1/2_ band (162.8 eV) can be assigned to S^2−^ in MoS_2_.^12^ In the C 1*s* spectrum (Figure 3d), the broader peak can be decomposed into a C—C/C=C bond of 284.8 eV, a C—O—C/S bond of 286.1 eV, as well as some other carbonated species bonds, respectively.[qv: 10d]

**Figure 3:**
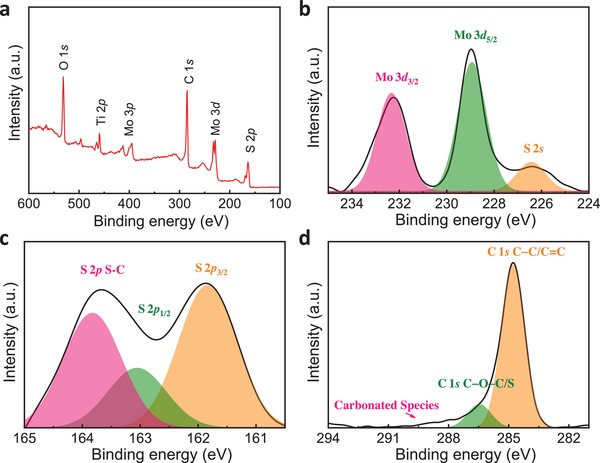
XPS analysis of NTO/MoS_2_‐C nanoarchitectures. a) Overall spectrum, and high‐resolution of b) Mo 3d, c) S 2p, d) C 1s spectra.

The heterogeneous NTO/MoS_2_‐C nanoarchitecture was then studied as the anode material of SIB, and metal sodium was chosen as the counter electrode and the reference electrode. **Figure** **4**a shows the first four consecutive cycling voltammograms (CV) curves of the anode from 0.05 to 3.00 V at a scan rate of 0.1 mV s^−1^. In the first sweep, the reductive peaks ≈0.2 and 1.1 V are mainly attributed to the irreversible electrochemical reactions, resulting the formation of solid electrolyte interface (SEI)[qv: 3d,6a] as well as a relatively low coulombic efficiency (CE) of about 61.8% (Table S1, Supporting Information). The reversible reductive peaks ≈0.7 V and below 0.5 V could be mainly attributed to the initial intercalation of Na^+^ into the MoS_2_ interlayer (Equation 1), and the conversion reaction from Na*_x_*MoS_2_ into metallic Mo nanoparticles and Na_2_S^13^ (Equation 2), respectively. In the reverse anodic sweep, a broad peak at ≈1.8 V corresponds to the oxidation from Mo to MoS_2_ (Equation 3),^14^ and a tiny peak at 2.25 V is associated with the desodiation of Na_2_S to S.^12^ It is not easy to distinguish the oxidation peak of NTO, owing to the relatively low current and the overlap of currents between MoS_2_ and NTO. However, based on the CV curves of pure NTO materials (Figure S8c, Supporting Information), it is obvious that the peaks at 0.2 and 0.6 V correspond to the redox couple of Ti^4+^/Ti^3+^ during sodiation/desodiation process (Equation 4).[qv: 3b] After the second scan, the peak position and peak shape of the CV curves were well maintained and overlapped, indicating an excellent electrochemical reaction reversibility in the electrochemical process. The reversible electrochemical reactions in the material can be expressed as follows:

(1) $${\mathrm{MoS}}_{2}+x{\mathrm{Na}}^{+}+xe^{-}\to {\mathrm{Na}}_{x}{\mathrm{MoS}}_{2}\left(\mathrm{above}\;0.5\;V\;\mathrm{vs}\;{\mathrm{Na/Na}}^{+}\right)$$

(2) $$\begin{array}{l} {\mathrm{Na}}_{x}{\mathrm{MoS}}_{2}+\left(4-x\right){\mathrm{Na}}^{+}+\left(4-x\right)e^{-}\to \mathrm{Mo}+2{\mathrm{Na}}_{2}S\\ \;\;\;\;\;\;\;\;\;\;\;\;\;\;\;\;\;\;\;\;\;\;\;\;\;\;\;\;\left(\mathrm{below}\;0.5\;V\;\mathrm{vs}\;{\mathrm{Na/Na}}^{+}\right) \end{array}$$

(3) $$\mathrm{Mo}+2{\mathrm{Na}}_{2}S\to {\mathrm{MoS}}_{2}+4{\mathrm{Na}}^{+}+4e^{-}$$

(4) $${\mathrm{Na}}_{2}{\mathrm{Ti}}_{9}O_{19}+y{\mathrm{Na}}^{+}+ye^{-}\to {\mathrm{Na}}_{2+y}{\mathrm{Ti}}_{9}O_{19}$$

**Figure 4:**
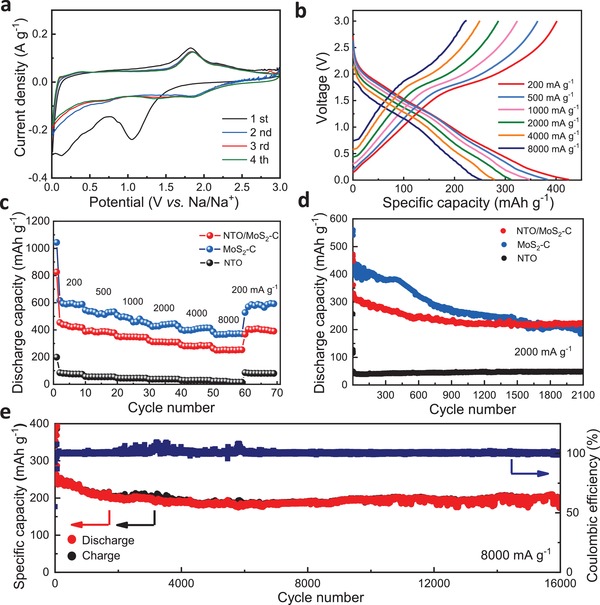
Electrochemical performance of NTO/MoS_2_‐C in SIBs. a) CV curves of NTO/MoS_2_‐C at a scan rate of 0.1 mV s^−1^. b) Galvanostatic discharge/charge profiles of NTO/MoS_2_‐C. Comparison of c) rate performances and d) cycling performances at 2000 mA g^−1^ among NTO/MoS_2_‐C, MoS_2_‐C, and NTO electrode materials. e) Cycling stability and coulombic efficiency of NTO/MoS_2_‐C at 8000 mA g^−1^ between 0.01 and 3.0 V.

Herein, the NTO/MoS_2_‐C, MoS_2_‐C, and NTO electrode materials (please see more details in Material preparation part and Figures S6 and S7, Supporting Information) were also tested for constant current charge and discharge at current densities of 200, 500, 1000, 2000, 4000, and 8000 mA g^−1^ (Figure 4b and Figure S9, Supporting Information). The charge/discharge curves of NTO/MoS_2_‐C maintained the same shape with a slight polarization, implying outstanding fast ionic and electronic transport properties (Figure 4b). At a relatively low current density (200 mA g^−1^), the NTO/MoS_2_‐C electrode material released a stable capacity of up to 425.5 mAh g^−1^. When the current density increased from 500 to 4000 mA g^−1^, the specific capacity of the material decreased from 383.6 to 348.4, 311.5, and 279.4 mAh g^−1^ (Figure 4c). Even at a large magnification of 8000 mA g^−1^, the specific capacity remained at 253.7 mAh g^−1^. When the current density was reduced to 100 mA g^−1^ again, the specific capacity of NTO/MoS_2_‐C can be restored to 408.8 mAh g^−1^, indicating that electrolyte permeation and activation of the electrode material have completed. As a comparison, when the current densities increased from 200 to 2000 mA g^−1^, the specific capacity of pure NTO electrode materials decreased from 76.5 to 37.2 mA g^−1^. At higher current density (4000 mA g^−1^), there is almost no capacity for pure NTO nanowires. MoS_2_‐C, however, exhibited a quite satisfactory specific capacity: from 594.1 to 366.5 mA g^−1^ when the current densities increased from 200 to 8000 mA g^−1^. This phenomenon could be attributed to higher content of the promoter MoS_2_ in MoS_2_‐C compared with that in NTO/MoS_2_‐C hybrid, together with higher specific surface area of MoS_2_‐C (259.9 m^2^ g^−1^) compared with that of NTO/MoS_2_‐C (176.0 m^2^ g^−1^) (Figure S10, Supporting Information). More MoS_2_ component and larger specific surface area give rise to relatively high capacity.

Capacity and cyclic stability are two critical factors to emulate the electrode materials. It has been demonstrated that structural synergetic advantages are favored for high‐rate capacity and long‐term cyclability. In order to exhibit the superiority of hybrid structure, cyclic stability of various samples was also carefully investigated. At the beginning of the test, the capacity of MoS_2_‐C nanospheres was superior to NTO/MoS_2_‐C and pure NTO nanowires. As shown in Figure 4d, the initial discharge specific capacity of MoS_2_‐C is 435.1 mA g^−1^ at 2000 mA g^−1^, which is also much higher than that of NTO/MoS_2_‐C. However, the specific capacity of MoS_2_‐C declines quickly after 500 cycles. In contrast, NTO/MoS_2_‐C shows more stable cyclability and even no capacity decay from 1000 to 2100 cycles. Compared to NTO/MoS_2_‐C and MoS_2_‐C, the capacity for NTO material shows an excellent cycling stability with almost no capacity decay for thousands of cycles, implying an ideal stable substrate for the high‐capacity promoter. It is proposed that the superiority of the design and protocol of NTO/MoS_2_‐C nanoarchitecture: the NTO substrate is quite useful for the whole architecture and NTO/MoS_2_‐C illustrates the best trade‐off for electrochemical performances in consideration of 1) low capacity but stable NTO substrate and 2) high capacity but unstable MoS_2_. As a result, the NTO/MoS_2_‐C electrode material exhibits an initial discharge specific capacity of up to 252.5 mAh g^−1^ at 8000 mA g^−1^ due to the uniform dispersion of the large‐capacity MoS_2_ nanosheets on 2D carbon conductive networks. The capacity was still maintained at 79.7% (capacity fade of only 0.0012% per cycle) after 16 000 super‐long cycles (Figure 4e). The morphology of the NTO/MoS_2_‐C electrode material after tens of thousands of cycles was also studied. The well‐preserved 3D heterogeneous structure proved it to be an ideal host during sodiation/desodiation reactions (Figure S12, Supporting Information). As a comparison, the specific capacities of MoS_2_‐C are relatively high for the first several cycles (≈300 mAh g^−1^) but decrease gradually for the next 5000 cycles with the fading rate of 0.013% per cycle (Figure S13, Supporting Information). By comparing the electrochemical performances with some recent reported works about NTO materials and MoS_2_‐based composites, NTO/MoS_2_‐C ranks as one of the best anodes for SIBs (Table S2, Supporting Information), reflecting the design superiority of 1D/2D–2D architecture proposed in this work.

The charge storage mechanism of three electrode materials was further investigated by sweep voltammetry, the current obeys a power‐law relationship with the sweep rate: *i* = *av^b^* (where *a* and *b* are adjustable parameters).^15^ **Figure** **5**a shows the log(*v*)–log(*i*) plot analysis of the three electrodes from CV curves at different current densities (Figure S8, Supporting Information). NTO substrate exhibits the relatively low *b* value (0.72), indicating that the electrochemical kinetic is mixed with surface‐controlled redox reaction and diffusion‐controlled redox reaction. For MoS_2_‐C, the *b* value is very close to 1 (0.97). It is a typical surface‐controlled redox process, which can make the most use of high‐capacity property of nano‐MoS_2_. The *b* value of NTO/MoS_2_‐C, as a result, shows intermediate value between 0.72 and 0.97, leading to the best cyclability and capacity combination. To have better understanding about the contribution of surface‐controlled redox current (*I*_c_) and diffusion‐controlled redox current (*I*_d_), the total peak current of one electrode *i* = *av^b^* can be described by the following equations: *i* = *I*_c_ + *I*_d_ = *k*_1_ *v* + *k*_2_ *v* ^1/2^, or *i v* ^−1/2^ = *k*_1_ *v* ^1/2^ + *k*_2_. Therefore, the coefficients *k*_1_ and *k*_2_ can be calculated by linear fitting of the cathode and anode peaks of *i v* ^−1/2^ versus *v* ^1/2^ (Figure 5b), and contribution ratio is shown in Figure 5c. As the scan rates change from 0.05 to 0.6 mV s^−1^, the contribution of pseudocapacity‐controlled current of anodic peak increase from 58.1%, 66.2%, 73.5%, 79.7% to 82.7%, respectively. It should be noted that for cathodic peak, NTO/MoS_2_‐C electrode is mainly controlled by pseudocapacitive current, even though at quite low scan rate of 0.05 mV s^−1^ (96%). This cathodic process could be regarded as a typical solid solution behavior. Such a solid solution electrochemical reaction is conducive to the super high‐rate capability due to the elimination of nucleation‐and‐growth kinetics of two‐phase transformations.^16^

**Figure 5:**
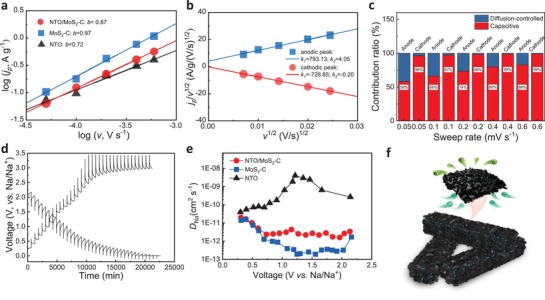
Electrochemical mechanism analysis of NTO/MoS_2_‐C in SIBs. a) *b* values analysis, b) *v* ^1/2^–*j*_p_/*v* ^1/2^ plot analysis, c) contribution ratio of the capacitive and diffusion‐controlled currents versus scan rate. d) GITT curves and e) comparison of Na^+^ diffusion coefficient (*D*_Na_) calculated from the GITT curves among NTO/MoS_2_‐C, MoS_2_‐C and NTO electrodes. f) Schematic diagram of the fast Na^+^ transfer behaviors of NTO/MoS_2_‐C in SIBs.

To investigate the electron conductivity among the three electrodes, electrochemical impedance spectroscopy (EIS) was conducted (Figure S14, Supporting Information). The charge‐transfer resistance (*R*_ct_, obtained from the semicircle at the high‐medium frequency region) of the NTO/MoS_2_‐C electrode is smaller than those of MoS_2_‐C and NTO, implying an improved electronic conductivity of the NTO/MoS_2_‐C with novel 1D/2D–2D hierarchical architectures. To further illustrate the chemical diffusion coefficient of Na^+^, galvanostatic intermittent titration technique (GITT) was conducted, according to which the sodium diffusion coefficient (*D*_Na_) can be calculated as follows:^17^

(5) $$D_{\mathrm{Na}}=\frac{4}{\pi }{\left(\frac{V_{M}}{A{\mathrm{FZ}}_{i}}\right)}^{2}{\left[I_{0}\left(\frac{dE}{d\delta }\right)/\left(\frac{dE}{d\sqrt{t}}\right)\right]}^{2},\;\;\;t<<\frac{L^{2}}{D_{\mathrm{Na}}}$$

where *D*_Na_ (cm^2^ s^−1^) is the chemical diffusion coefficient of the mobile material, *V*_M_ is the molar volume of the active material (31.63 cm^3^ mol^−1^ for MoS_2_), and *A* (cm^2^) is the total contact area between the electrolyte and the electrode. *F* is the Faraday constant, *Z_i_* is the valence of the substance, *I*_0_ (A) is the applied constant current, (d*E* /dδ) is determined by the quasi‐equilibrium potential against the stoichiometry concentration of Na^+^ in one electrode (*E* is electrode voltage, δ is the deviation from the stoichiometric ratio), *t* is the time during constant current pulse and *L* (cm) is the thickness of the electrode. Based on the above equation, the Na^+^ diffusion coefficient calculated on the GITT curves (Figure 5d and Figure S15, Supporting Information) is a function of the potential at the initial open‐circuit cell potential, as shown in Figure S16 (Supporting Information) and Figure 5e. During the discharge process, *D*_Na_ fluctuates between 10^−11^–10^−12^ cm^2^ s^−1^; and during the charge process, *D*_Na_ shows about one order of magnitude enhancement below 1.0 V but decreases rapidly to ≈10^−13^ cm^2^ s^−1^ between 1.5–2.0 V, and finally recovers to the same order of magnitude as that of discharge process (≈10^−12^ cm^2^ s^−1^). The phenomenon may be caused by the differences of contribution ratios of the diffusion‐controlled current during charging and discharging. Just as mentioned above, the anodic (charging) process of NTO/MoS_2_‐C electrode is mixed by pseudocapacitive current and diffusion‐controlled current compared with mainly diffusion‐controlled current of cathodic (discharging) process. The diffusion‐controlled current is obviously illustrated between 1.5 and 2.0 V, in which region the nucleation‐and‐growth kinetics of two‐phase transformations reduce the ionic diffusion coefficient. This interesting result shows that it is easier to achieve a satisfactory capacity during the discharging process in a half cell (the charging process in a full cell), thus making the NTO/MoS_2_‐C a very promising anode for fast charging batteries. Because of the layered structure of NTO with large interlayer spacing for ionic transport, the *D*_Na_ of NTO substrate show about one order of magnitude higher than those of MoS_2_‐C, especially in the voltage range of 1–2 V (Figure 5e). Therefore, it is not difficult to understand that NTO/MoS_2_‐C exhibits a trend of diffusion enhancement compared with MoS_2_‐C. This result illustrates that the presence of NTO nanowires in NTO/MoS_2_‐C can not only act as a robust substrate with excellent stability, but also can be regarded as an ideal ionic conductor for boosting ion migration in the hierarchical architectures as well (Figure 5f). Lastly, sodium ion full cells were further assembled and measured using carbon‐coated Na_3_V_2_(PO_4_)_3_ materials as the cathode and NTO/MoS_2_‐C as the anode. As shown in Figure S17 (Supporting Information), due to the irreversible reactions in both electrodes together with limited sodium ions in full cells, the electrochemical performances are not as satisfactory as those in half cells, especially for the beginning several cycles. However, the high‐rate capacity and cyclability became relatively stable after those cycles. The results not only demonstrate the fast‐electrochemical kinetics of NTO/MoS_2_‐C as an anode for SIBs, but also emphasize the significance of high CE as well as matched working potential of one electrode when serving in full batteries.

In summary, a heterogeneous architecture of 1D/2D‐2D NTO/MoS_2_‐C has been synthesized and demonstrated as an advanced electrode with high‐rate and super‐durable performance in SIBs. The as‐prepared NTO/MoS_2_‐C hybrid possesses several structural advantages. First, 1D titanium‐based nanowires not only can play as an important role in structural stability, but also can enhance the ionic diffusion conductivity and alleviate stress and volume expansion caused by ion insertion/extraction; in addition, 2D conducive carbon nanonetworks can reduce the agglomeration of 2D MoS_2_ nanosheets during charge and discharge and improve electronic conductivity, ionic diffusion coefficient as well as enhance the wettability of electrode materials; moreover, external 2D MoS_2_ nanosheets can greatly increase the specific capacity. Benefitting from the synergistic effects of various structures, the resulting sample exhibited excellent electrochemical performance in SIBs. At a relatively low current density (200 mA g^−1^), the NTO/MoS_2_‐C electrode materials released a stable specific capacity of up to 425.5 mAh g^−1^. Even at a large rate of 8000 mA g^−1^, the NTO/MoS_2_‐C composite retained a reversible specific capacity of 201.1 mAh g^−1^ after 16 000 cycles. The charge storage mechanism and chemical diffusion coefficient analysis imply that NTO/MoS_2_‐C can be an ideal and promising host for high‐power battery systems. This work highlights the design protocol and synthesis strategy of the hierarchical architecture and exhibits their broad prospects in energy storage applications.

## Conflict of Interest

The authors declare no conflict of interest.

## Supporting information

Supplementary
