# Supplementary material for: MoS2‐Coupled Carbon Nanosheets Encapsulated on Sodium Titanate Nanowires as Super‐Durable Anode Material for Sodium‐Ion Batteries
Source: Adv Sci (Weinh). 2019 Mar 22;6(10):1900028. doi: 10.1002/advs.201900028 (PMC6523371; doi:10.1002/advs.201900028)
Supplement: Supplementary file 1 — Supplementary [file ADVS-6-1900028-s001.pdf]

Copyright WILEY-VCH Verlag GmbH & Co. KGaA, 69469 Weinheim, Germany, 2019.

ADVANCED SCIENCE
Open Access

Supporting Information

for *Adv. Sci.,* DOI: 10.1002/advs.201900028

MoS_2-Coupled Carbon Nanosheets Encapsulated on Sodium Titanate Nanowires as Super-Durable Anode Material for Sodium-Ion Batteries

Shitong Wang, Fangjun Cao, Yutong Li, Zhongtai Zhang, Daming Zhou, Yong Yang, and Zilong Tang**

Copyright WILEY-VCH Verlag GmbH & Co. KGaA, 69469 Weinheim, Germany, 2019.

Supporting Information

MoS_2-coupled Carbon Nanosheets Encapsulated on Sodium Titanate Nanowires as Super-durable Anode Material for Sodium-Ion Battery

Shitong Wang, Fangjun Cao, Yutong Li, Zhongtai Zhang, Daming Zhou, Yong Yang* and Zilong Tang*

Dr. S. Wang, Ms. Y. Li, Prof. Z. Zhang, Prof. Z. Tang
State Key Lab of New Ceramics and Fine Processing, School of Materials Science and Engineering, Tsinghua University, Beijing 100084, China
Dr. S. Wang
Department of Nuclear Science and Engineering, Massachusetts Institute of Technology, Cambridge, Massachusetts 02139, USA
Prof. D. Zhou
School of Astronautics, Northwestern Polytechnical University, Xi'an, Shanxi 710072, China
Dr. F. Cao, Prof. Y. Yang
State Key Lab of Solidification Processing Center of Advanced Lubrication and Seal Materials, Northwestern Polytechnical University, Xi'an, Shanxi 710072, China

Supplementary figures S1~S17
Supplementary tables S1~S2
Supplementary references S1~S17

Experimental Section

Material Preparation. The sodium titanate precursor was first synthesized via a typical hydrothemal treatment, that is, a certain amount of anatase TiO_2 was added into 15 M NaOH solution before transferring in Teflon-lined stainless-steel autoclave. After hydrothemal reactions at 180 °C for 48 h, the white product was obtained by washing and filtration. Second, 10 mg $Na_2Ti_2O_5 \cdot H_2O$ powder, a certain amount of ammonium molybdate ($(NH_4)_6Mo_7O_{24}$) and dopamine hydrochloride (dopamine·HCl) were dispersed and dissolved in the mixture solution of distilled water and ethanol (volume ratio 1:2). Finally, after adding ammonium hydroxide (20 μL) into the above solution, the obtained orange red $Na_2Ti_2O_5 \cdot H_2O$/Mo-polydopamine product was stirred continuously for 6 h. After heat treatment between $Na_2Ti_2O_5 \cdot H_2O$/Mo-PDA powder and a certain amount of sulphur at 600 °C for 2 h under N_2, $Na_2Ti_2O_5 \cdot H_2O$/Mo-PDA was transferred to $Na_2Ti_9O_{19}/MoS_2$-C. For comparison, MoS_2-carbon hybrid (denoted as MoS_2-C) was obtained without adding $Na_2Ti_2O_5 \cdot H_2O$; pure $Na_2Ti_9O_{19}$ nanowires (denoted as NTO) was prepared without adding ammonium molybdate and dopamine hydrochloride, while all the other experimental procedures remained unchanged. $Na_3V_2(PO_4)_3$ cathode was synthesized by simple hydrothermal reactions of V_2O_5, $H_2C_2O_4 \cdot 2H_2O$ and NaH_2PO_4 followed by glucose carbon coating process, see more details in reference [S1].

Material Characterization. The phases of the as-prepared samples were tested on a Rigaku D/Max-B X with Cu Kα radiation (λ=1.5418 Å) for X-ray diffraction (XRD). The morphology and size of the samples were studied via scanning electron microscope (SEM, MERLIN VP Compact) and transmission electron microscopy (TEM, Hitachi-HT7700). High resolution transmission electron microscopy (HRTEM) and high-angle annular dark-field (HAADF)-scanning transition electron microscopy (STEM) were conducted on JEM-2100F equipped with energy dispersive spectrometer (EDS). The Raman spectrum was conducted on a HR800 Raman spectrometer (HORIBA) using 633 nm line of a helium-neon as the excitation beam. X-ray photoelectron spectroscopy (XPS) analyses was conducted on Escalab 250XI system (Thermo Fisher Scientific). The content of elements was determined through inductively coupled plasma mass spectroscopy (ICP-MS) analysis (Thermo Fisher Scientific). The specific surface area was obtained using an automated vapor sorption analyzer (Autosorb-iQ2-MP, Quanta Chrome) at 77.4 K under vacuum by Brunauer-Emmett-Teller (BET) method.

Electrochemical Measurements. For the electrochemical measurements, 70 wt% active materials, 20 wt% Super P, and 10 wt% poly (vinylidene fluoride) binder were first added in N-methyl-2-pyrrolidinone (NMP) solvent. The well-mixed slurry was casted on a Cu foil (for NTO/MoS_2-C, MoS_2-C and NTO anodes) or an Al foil (for $Na_3V_2(PO_4)_3$ cathode), then dried at 110 °C in a vacuum overnight. For half cells, sodium foil was chosen as the counter electrode and for full cells, $Na_3V_2(PO_4)_3$ was chosen as the cathode. A glass fiber (Whatman) was used as the separator. The electrolyte was composed of 1 M $NaClO_4$ dissolved in a mixture of ethylene carbonate-dimethyl carbonate (1:1 in volume) with 5 wt% fluoroethylene carbonate additives. The mass loading of NTO/MoS_2-C, MoS_2-C and NTO electrodes in this work is controlled around 1.0 mg cm^{-2}. The mass loading of $Na_3V_2(PO_4)_3$ cathode is about 4.5 mg cm^{-2}, providing ~50% excessive capacity compared with NTO/MoS_2-C anode. Cyclic voltammetry (CV) and electrochemical impedance spectroscopy (EIS, 10~100 kHz with a perturbation of 5 mV) were performed by IM6 (Bas-Zahner, Germany) electrochemical workstation. Before the galvanostatic intermittent titration technique (GITT) measurement, the cell was cycled for 5 cycles at 100 mA g^{-1}. Then the measurements were performed by applying a current density of 50 mA g^{-1} followed by a relaxation period of 10 h. All tests were measured on a LAND 2001A Cell test system between 0.05~3.00 V (for half cells) and 0.05~4.00 V (for full cells) at ambient temperature.

Supplementary Figures

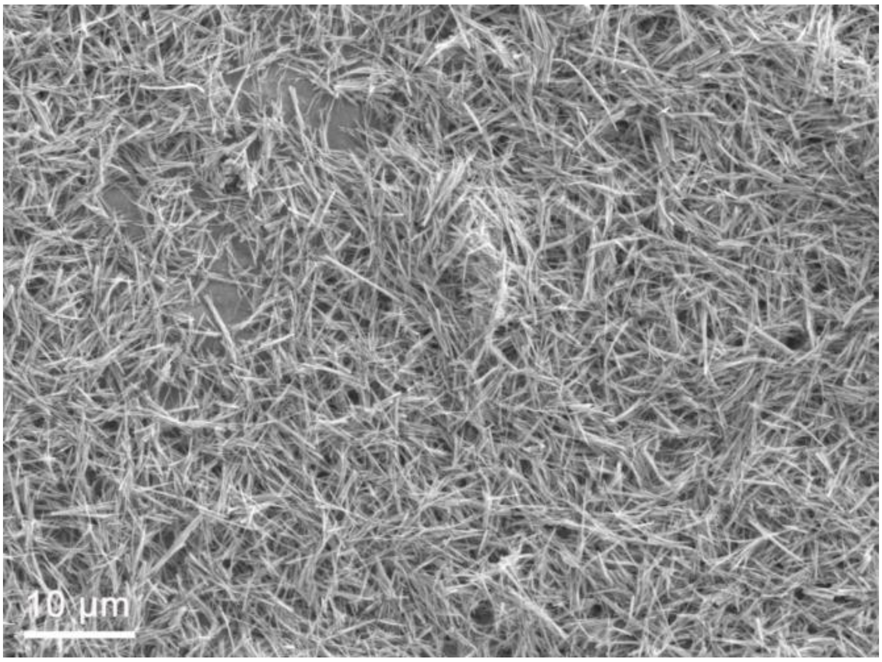

Figure S1. SEM image of $Na_2Ti_2O_5 \cdot H_2O$ nanowires.

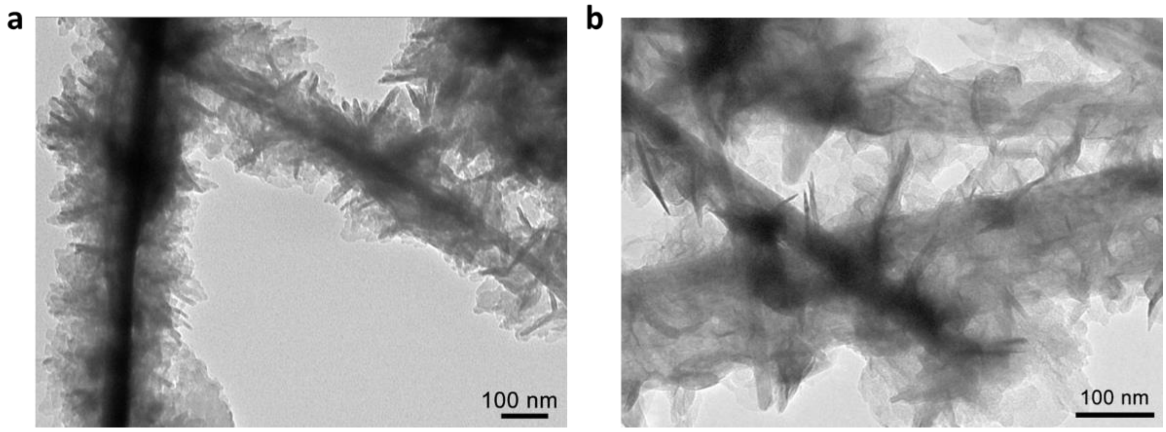

Figure S2. (a-b) TEM images of heterogeneous architectures of $Na_2Ti_2O_5{\cdot}H_2O$/Mo-PDA.

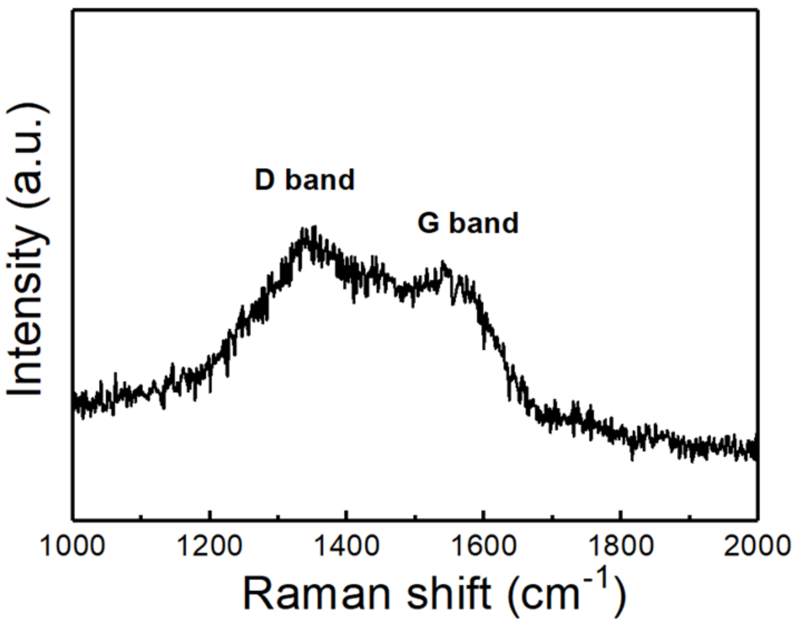

Figure S3. Raman spectrum of NTO/MoS_2-C composite.

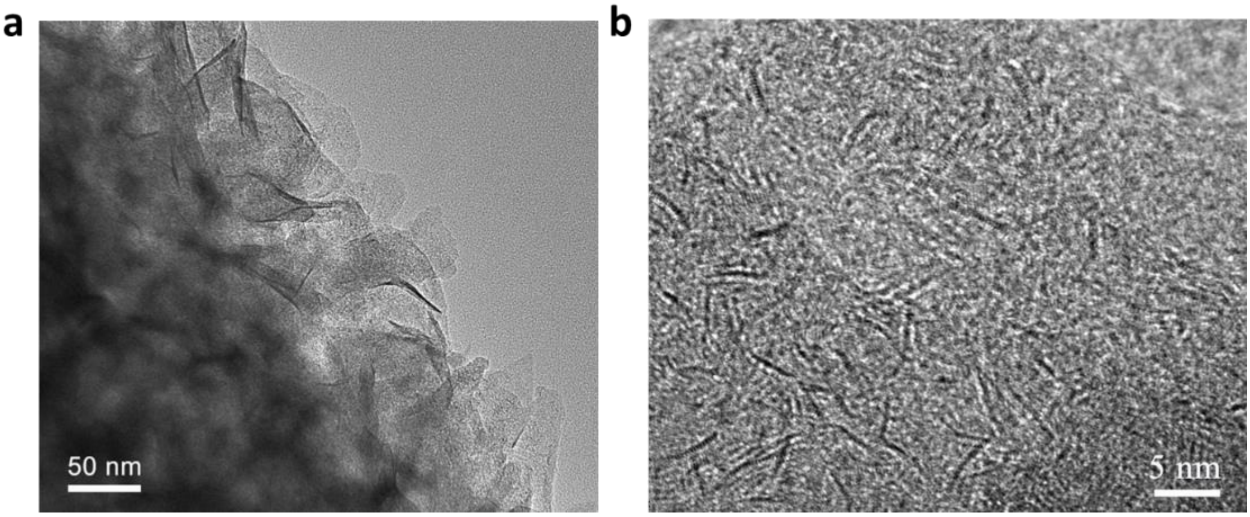

Figure S4. (a-b) HRTEM analysis of MoS_2 in NTO/MoS_2-C composite.

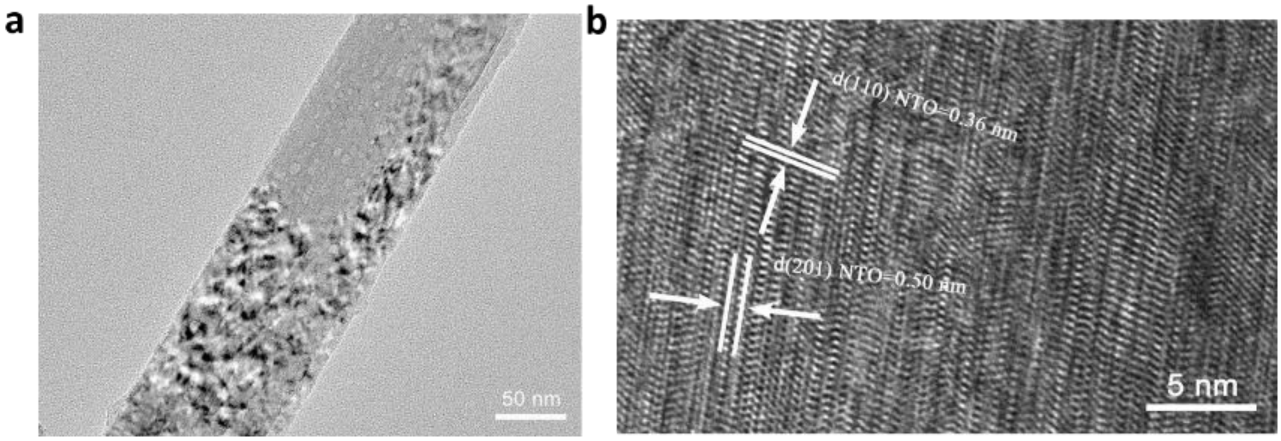

Figure S5. (a-b) HRTEM analysis of NTO nanowires.

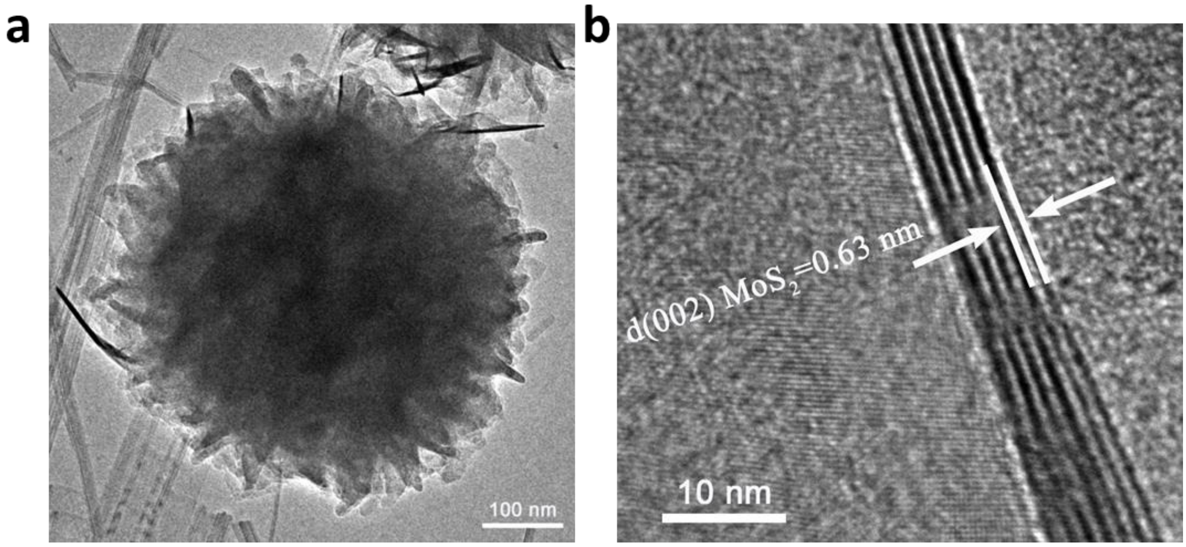

Figure S6. HRTEM analysis of MoS_2-C hybrid.

The MoS_2-C materials illustrate a nanospheres morphology (a diameter of 400~500 nm) composed of aligned MoS_2-carbon coupled interface nanosheets (**Figure S6a**), and the interplanar spacing of MoS_2 nanosheets is 0.63 nm corresponds to the (002) crystal planes of MoS_2 (**Figure S6b**).

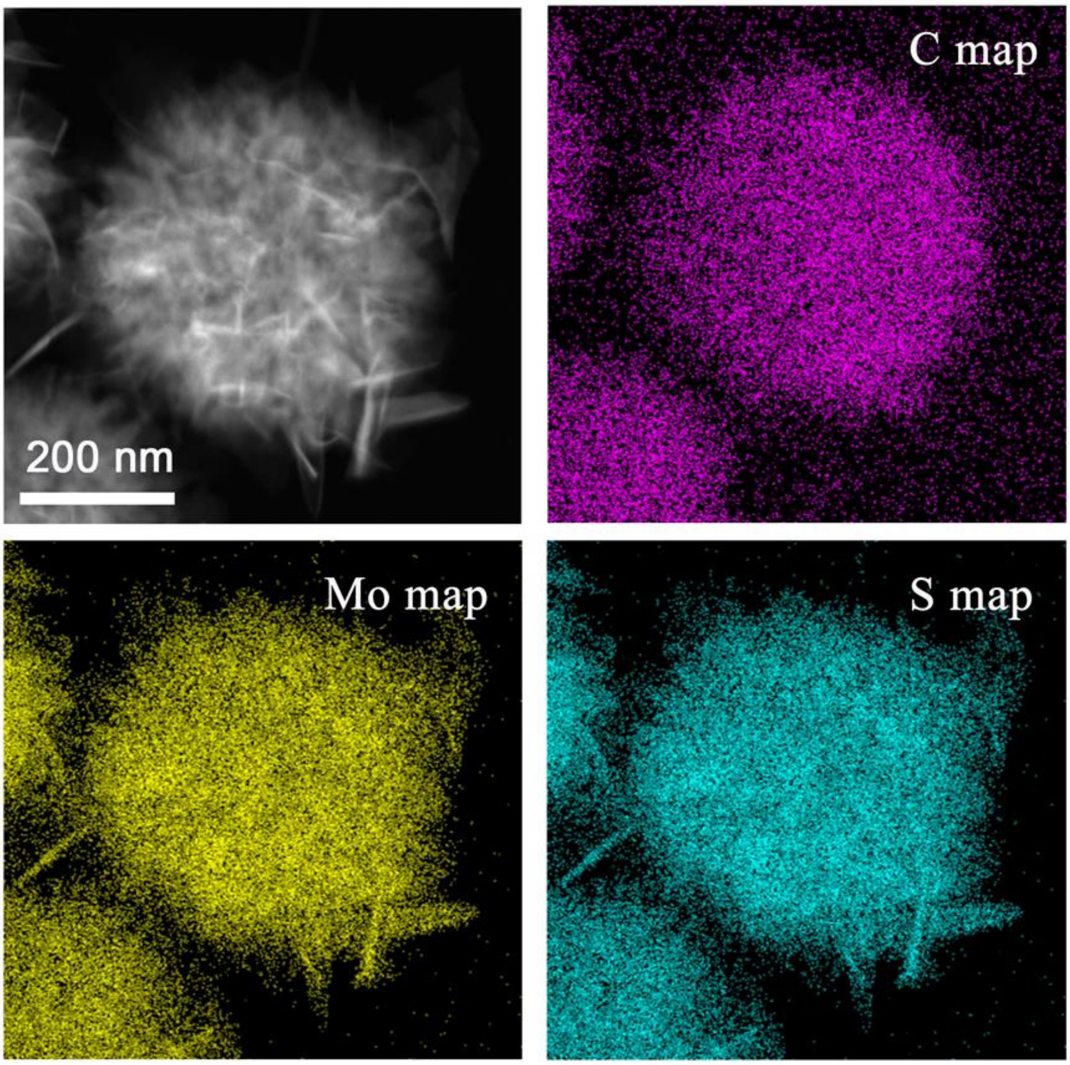

Figure S7. HRTEM analysis of MoS_2-C materials. The corresponding EDS mapping analysis of the distribution of Mo, C and S elements demonstrate that the MoS_2 nanosheets are uniformly distributed in the carbon nanosheets layer.

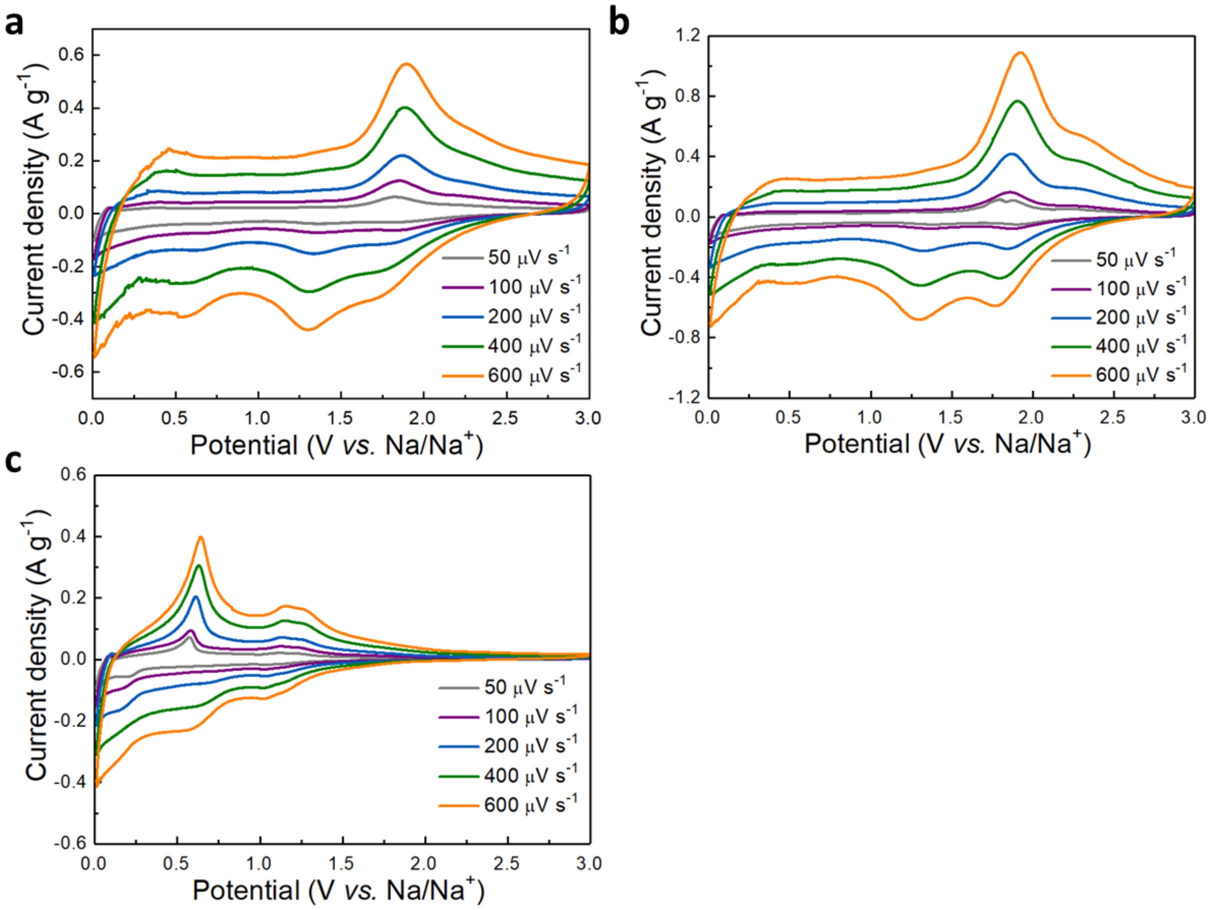

Figure S8. CV curves of (a) NTO/MoS_2-C, (b) MoS_2-C and (c) NTO electrodes at different scan rates.

The main redox currents of NTO/MoS_2-C can be attributed to those of MoS_2-C, which is consistent with the result of rate performances in **Figure 4c** (much more specific capacity of MoS_2-C compared with that of NTO electrode).

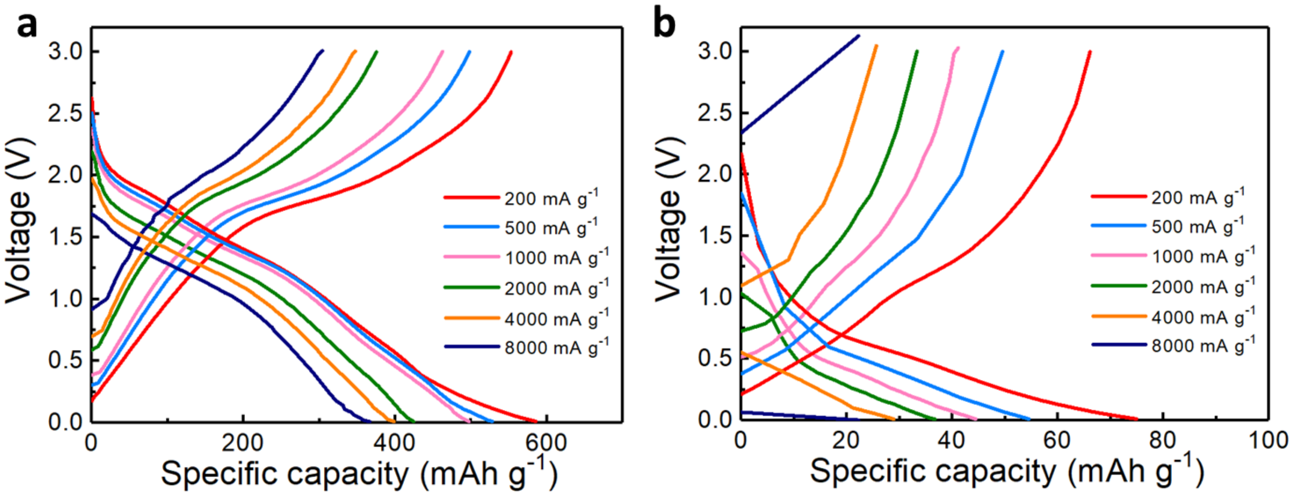

Figure S9. Galvanostatic discharge/charge profiles of (a) MoS_2-C and (b) NTO materials.

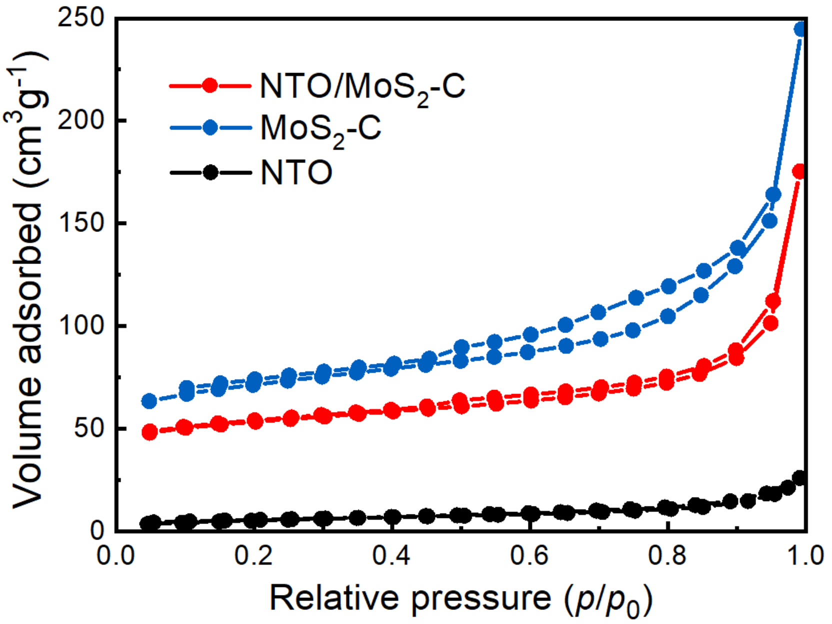

Figure S10. N_2 adsorption-desorption analysis of NTO/MoS_2-C, MoS_2-C and NTO materials.

The results show that NTO/MoS_2-C has a much higher specific surface area (176.0 m^2 g^{-1}) than that of NTO (23.6 m^2 g^{-1}). This is attributed to the successful growth of 2D heterojunction nanosheets on 1D nanowires. However, the MoS_2-C exhibited the highest specific surface area of 259.9 m^2 g^{-1} among them.

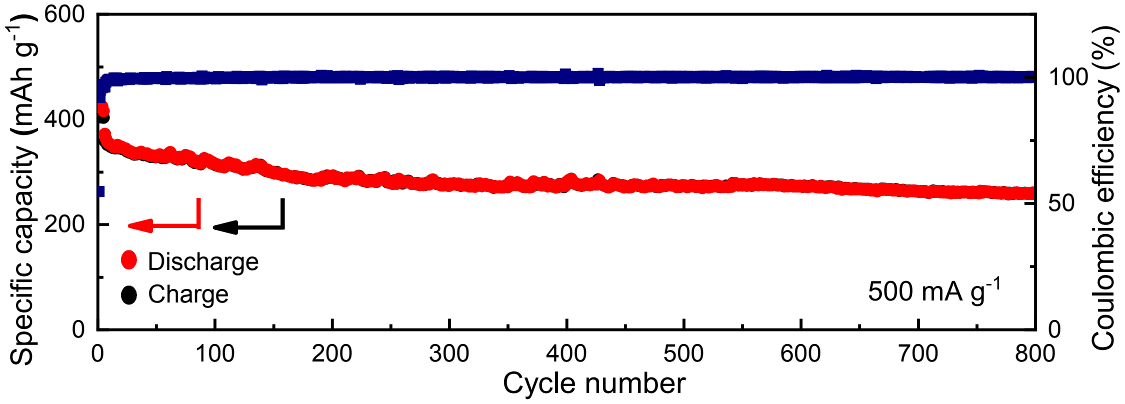

Figure S11. Cycling stability and Coulombic efficiency of NTO/MoS_2-C at 500 mA g^{-1}.

Because side reactions (such as electrolyte decomposition) at high current density may have a negligible contribution to the total current compared to that of the active material's redox reaction, while they might not be the case at low rate. Therefore, cycling performance at low current density (500 mA g^{-1}) was performed. It can deliver a specific capacity of 372.1 mAh g^{-1} and maintain more than 800 cycles with 72.9% capacity retention and almost 100% Coulombic efficiency, demonstrating a relatively stability of the electrode.

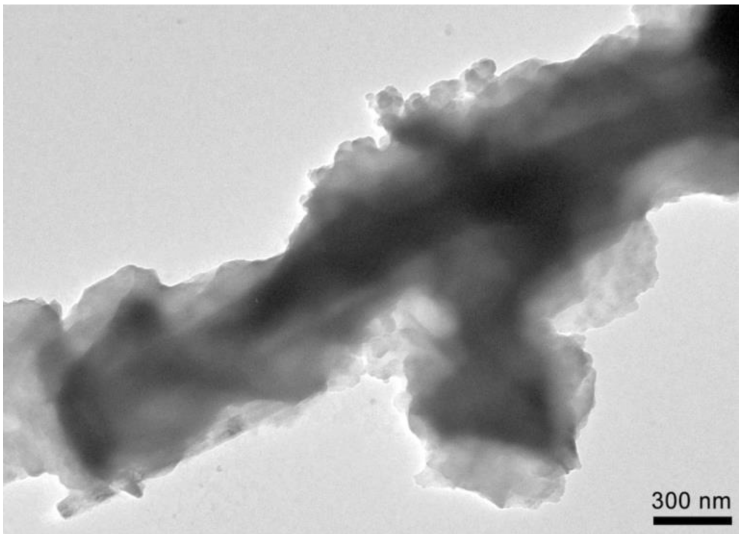

Figure S12. TEM analysis of NTO/MoS_2-C after 16000 cycles.

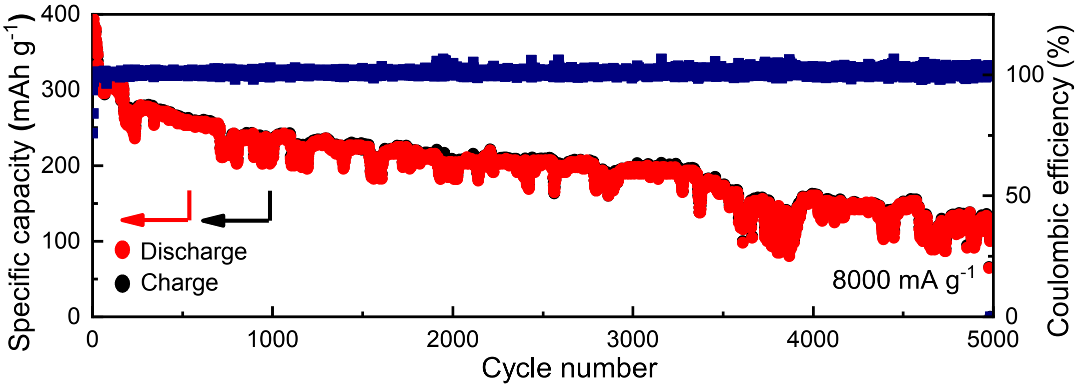

Figure S13. Cycling stability and Coulombic efficiency of MoS_2-C at 8000 mA g^{-1}.

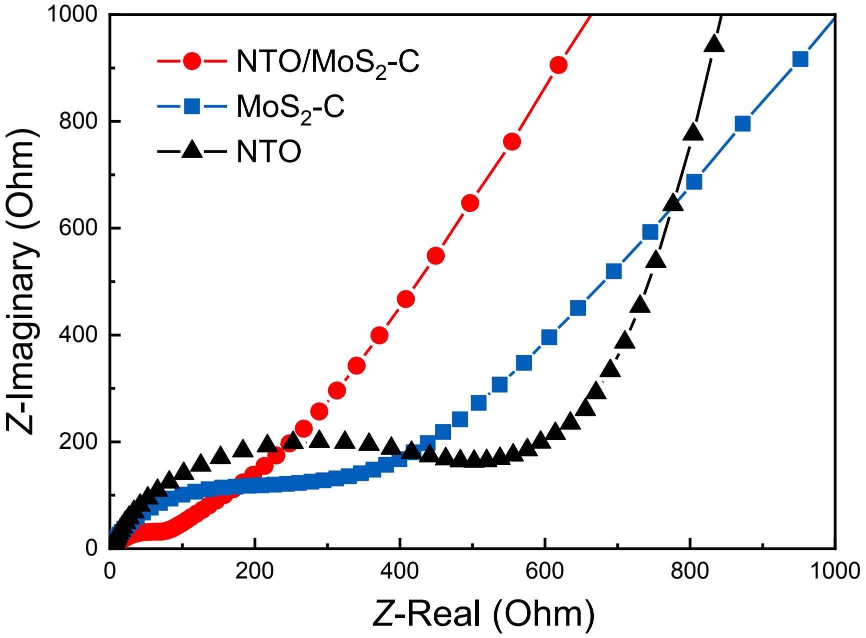

Figure S14. EIS analysis for NTO/MoS_2-C, MoS_2-C and NTO electrodes.

The electron conductivity of the as-synthesized electrodes can be investigated by electrochemical impedance spectroscopy (EIS). The semicircle at the high-medium frequency region can be ascribed to the charge-transfer resistance (R_{ct}). It is evident that the R_{ct} of the NTO/MoS_2-C electrode is smaller than those of MoS_2-C and NTO, implying that the NTO/MoS_2-C with novel 1D/2D-2D hierarchical architectures could exhibit the improved electronic conductivity.

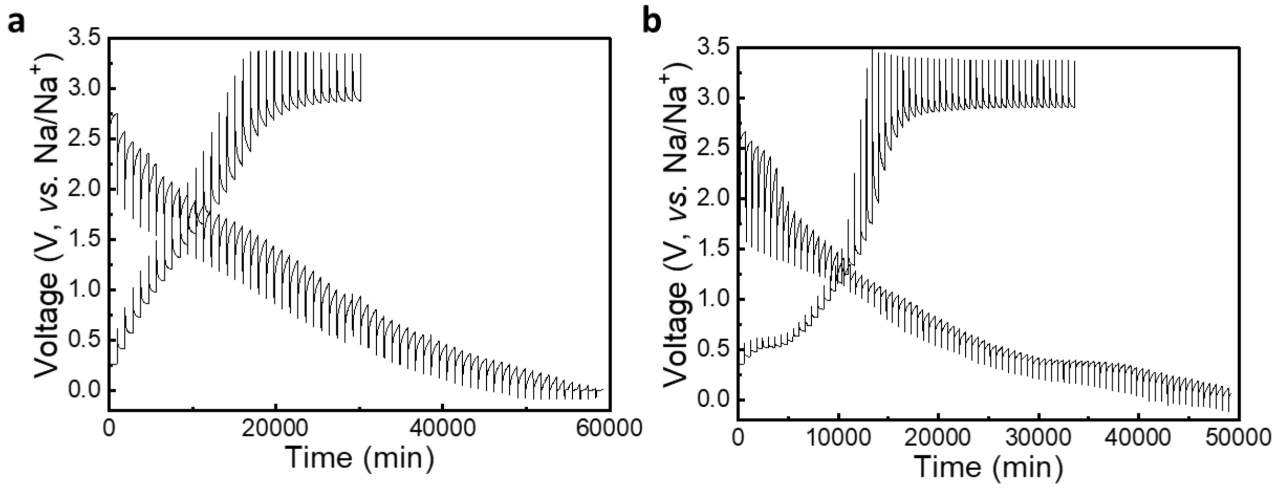

Figure S15. Galvanostatic intermittent titration technique (GITT) curves of (a) MoS_2-C and (b) NTO electrodes.

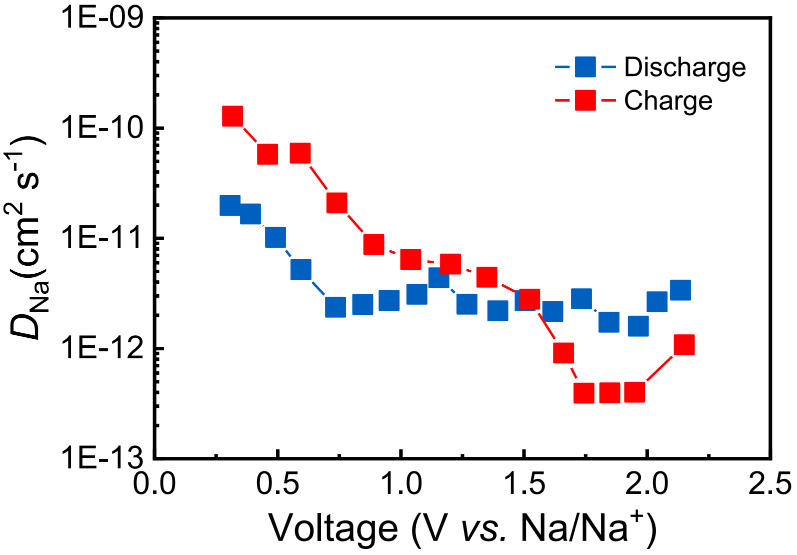

Figure S16. Na^+ diffusion coefficient (D_{Na}) of NTO/MoS_2-C calculated from GITT curves.

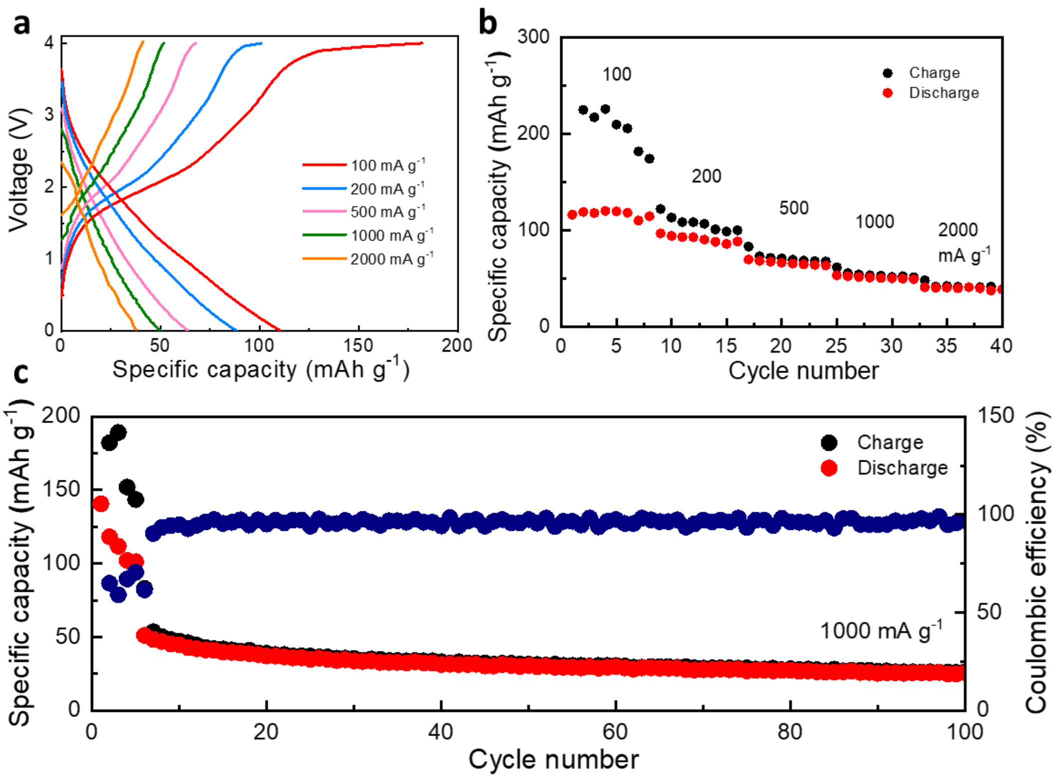

Figure S17. Electrochemical performance of full-cell testing using NTO/MoS_2-C as the anode and $Na_3V_2(PO_4)_3$ as the cathode in SIBs. (a) Galvanostatic discharge/charge profiles and (b) rate performances from 100 to 2000 mA g^{-1}, (c) cycling performances and Coulombic efficiency at 1000 mA g^{-1} (after 5 cycles' activation at 100 mA g^{-1}).

With the increase of current densities from 100 to 2000 mA g^{-1}, the full cell can deliver specific capacities of 110.4, 88.3, 63.9, 50.4 and 37.8 mAh g^{-1}, respectively (**Figure S17a, b**). After activation for 5 cycles at 100 mA g^{-1}, the specific capacity can sustain for 100 cycles at high rate of 1000 mA g^{-1}, with relatively stable cyclability and nearly 100% CE (**Figure S17c**).

Supplementary Tables

Table S1. The initial Coulombic efficiencies of NTO/MoS_2-C, MoS_2-C and NTO materials at 100 mA g^{-1}.

Materials	Initial Coulombic efficiency (%)
**NTO/MoS_2-C**	61.8
**MoS_2-C**	58.9
**NTO**	40.2

Table S2. Comparison of this work with previously reported sodium titanate materials, high-capacity MoS_2 materials as well as their hierarchical composites. Their electrode compositions are listed using the mass ratio of active materials: conductive carbon: binder.

Materials	Electrode compositions	Loading density (mg cm^{-2})	Capacity (retention) after cycles (at current density)	Refs
**1D/2D-2D hierarchical NTO/MoS_2-C nanoachitecture**	**70:20:10**	**~1.0**	**201, 16000 cycles (8000 mA g^{-1})**	**This work**
**Sodium titanate nanowire@ rGO**	100:0:0	1~1.5	~150, 3000 cycles (2 C)	[S2]
**$Na_2Ti_3O_7$@N-doped carbon hollow spheres**	60:30:10	1	68, 1000 cycles (8800 mA g^{-1})	[S3]
**Hydrogenation driven conductive $Na_2Ti_3O_7$ nanoarrays**	100:0:0	~0.3	65, 10000 cycles (6200 mA g^{-1})	[S4]
**$Na_2Ti_3O_7$ nanosheet arrays/ carbon textiles**	100:0:0	1	130, 1000 cycles (1000 mA g^{-1})	[S5]
**$Na_2Ti_6O_{13}$ nanorods**	75:15:10	0.8	129, 800 cycles (400 mA g^{-1})	[S6]
**Spider web-like $Na_2Ti_3O_7$ nanotubes**	100:0:0	N/A	107, 500 cycles (500 mA g^{-1})	[S7]
**$Na_2Ti_6O_{13}$-graphite composite**	70:20:10	1	~15, 5000 cycles (20 C)	[S8]
**MoS_2/carbon fiber hybrid**	80:10:10	N/A	333, 1000 cycles (1000 mA g^{-1})	[S9]
**Porous MoS_2/carbon spheres on MWCNT**	100:0:15.8	1.2	416, 1000 cycles (2000 mA g^{-1})	[S10]
**Super wide-interlayer spacing MoS_2/carbon fiber**	100:0:0	1	127, 3000 cycles (5000 mA g^{-1})	[S11]
**Vertically aligned MoS_2 nanosheets on exfoliated graphene**	70:15:15	1	509, 200 cycles (1000 mA g^{-1})	[S12]
**MoS_2/graphene nanosheets**	80:10:10	1.5~2.0	421, 250 cycles (300 mA g^{-1})	[S13]
**MoS_2 nanosheets@carbon nanowall array on carbon cloth**	100:0:0	1	265, 1000 cycles (1000 mA g^{-1})	[S14]
**1T MoS_2 sandwich grown on graphene tube**	100:0:0	0.5	313, 200 cycles (50 mA g^{-1})	[S15]
**MoS_2 nanosheets percolated with carbon nanotubes**	90:0:10	15	~315, 1000 cycles (500 mA g^{-1})	[S16]
**Ultrathin MoS_2 nanosheets**	70:20:10	1.2	251, 100 cycles (320 mA g^{-1})	[S17]

Supplementary References

[S1] W. Ren, Z. Zheng, C. Xu, C. Niu, Q. Wei, Q. An, K. Zhao, M. Yan, M. Qin and L. Mai, Nano Energy **2016**, 25, 145.

[S2] N. Q. Tran, T. A. Le and H. Lee, J. Mater. Chem. A **2018**, 6, 17495.

[S3] F. Xie, L. Zhang, D. Su, M. Jaroniec and S. Z. Qiao, Adv. Mater. **2017**, 29, 1700989.

[S4] S. Fu, J. Ni, Y. Xu, Q. Zhang and L. Li, Nano Lett. **2016**, 16, 4544.

[S5] S. Dong, L. Shen, H. Li, G. Pang, H. Dou and X. Zhang, Adv. Funct. Mater. **2016**, 26, 3703.

[S6] K. Cao, L. Jiao, W. K. Pang, H. Liu, T. Zhou, Z. Guo, Y. Wang and H. Yuan, Small **2016**, 12, 2991.

[S7] Y. Zhang, L. Guo and S. Yang, Chem Commun (Camb) **2014**, 50, 14029.

[S8] A. Rudola, K. Saravanan, S. Devaraj, H. Gong and P. Balaya, Chem Commun (Camb) **2013**, 49, 7451.

[S9] C. Cui, Z. Wei, J. Xu, Y. Zhang, S. Liu, H. Liu, M. Mao, S. Wang, J. Ma and S. Dou, Energy Storage Materials **2018**, 15, 22.

[S10] B. Chen, H. Lu, J. Zhou, C. Ye, C. Shi, N. Zhao and S.-Z. Qiao, Adv. Energy Mater. **2018**, 1702909.

[S11] C. Zhao, C. Yu, M. Zhang, Q. Sun, S. Li, M. Norouzi Banis, X. Han, Q. Dong, J. Yang, G. Wang, X. Sun and J. Qiu, Nano Energy **2017**, 41, 66.

[S12] G. Wang, J. Zhang, S. Yang, F. Wang, X. Zhuang, K. Müllen and X. Feng, Adv. Energy Mater. **2017**, 1702254.

[S13] D. Sun, D. Ye, P. Liu, Y. Tang, J. Guo, L. Wang and H. Wang, Adv. Energy Mater. **2017**, 1702383.

[S14] W. Ren, H. Zhang, C. Guan and C. Cheng, Adv. Funct. Mater. **2017**, 27, 1702116.

[S15] X. Geng, Y. Jiao, Y. Han, A. Mukhopadhyay, L. Yang and H. Zhu, Adv. Funct. Mater. **2017**, 1702998.

[S16] Y. Liu, X. He, D. Hanlon, A. Harvey, J. N. Coleman and Y. Li, ACS Nano **2016**, 10, 8821.

[S17] D. Su, S. Dou and G. Wang, Adv. Energy Mater. **2015**, 5, 1401205.
